# Adaptability, Interdisciplinarity, Engageability: Critical Reflections on Green Social Work Teaching and Training

**DOI:** 10.3390/healthcare10071245

**Published:** 2022-07-04

**Authors:** Haorui Wu, Meredith Greig

**Affiliations:** School of Social Work, Dalhousie University, Halifax, NS B3H 4R2, Canada; mr546260@dal.ca

**Keywords:** green social work, climate change, disasters, diverse crises, curriculum and professional training, critical reflection, micro-, mezzo-, macro-level interventions, health and well-being

## Abstract

The upward tendencies of global climate change, disasters, and other diverse crises have been urgently calling for green social work (GSW) interventions which engage a holistic approach to explore diverse societal dimensions’ compounded influences on inhabitants’ individual and collective health and well-being in disaster settings. Though globally gaining more attention, GSW has been slow to develop in the Canadian social work curriculum and professional training. This deficit jeopardizes integrating environmental and climate justice and sustainability in social work research and practice in Canada. In response to this pedagogical inadequacy, this article employs a critical reflection approach to examine two authors’ two-academic-year teaching–learning and supervision-training experiences of GSW-specific in-class and field education in a Master of Social Work program. The content analysis illustrates three essential components for GSW-specific teaching and training, namely adaptability, interdisciplinarity, and engageability. These components enhance the prospective social workers’ micro-, mezzo-, and macro-level practices to better support individuals, families, and communities affected by extreme events and promote their health and well-being in disaster and non-disaster scenarios. These GSW-specific pedagogies shed light on the fact that integrading climate change, disasters, and diverse crises in pedagogical innovations should be encouraged beyond the social work profession. A multidisciplinary multi-stakeholder engagement approach would comprehensively investigate and evaluate the essential components and evidence-based strategies that better serve inhabitants and promote resilience and sustainability.

## 1. Introduction

Promoting individual and collective health and well-being is one of the core mandates for social work research and practice engaging at the individual, family, community, and societal levels [1]. In 2012, Dominelli initiated the green social work (GSW) framework under the general social work umbrella, which contextualizes social work interventions in the global context of climate change, disasters, and other diverse crises [2]. GSW aims to enhance social workers’ holistic understanding of various societal dimensions that jointly shape human behaviors and impact their health and well-being, individually and collectively, in disaster settings [2]. Despite gaining recent attention, GSW has been slow to develop in the social work curriculum and professional training in Canadian social work education and practice [3]. This deficit has jeopardized the integration of environmental justice and sustainability into social work research and practice in the Canadian context and delayed Canadian social work professionals’ collaboration with their international peers from diverse backgrounds in addressing one of the most urgent issues of extreme events that threaten global sustainability [4]. These impacts eventually weaken the social workers’ advocacy in the domestic and international realms of social justice, human rights, health, and well-being.

Over the two-year academic period of 2020–2022, Canada experienced horrific disasters (e.g., COVID-19, floods, and mass shootings). This multi-disaster societal context has encouraged the first author to engage GSW in social work curriculum and professional training, forging two types of professional relationships with the second author, namely an instructor–student interplay (in-class education, 2020–2021) and a supervisor–trainee relationship (research-based field education, 2021–2022). Throughout this two-year interaction, the two authors frequently applied the critical reflection approach, which is an essential pedagogical instrument in health and social care disciplines [5], to integrate GSW components into the social work teaching-training process, with particular attention to compounded influences of extreme events on individual and collective health and well-being at different levels. These critical reflections, as well as third-party feedback (e.g., students’ evaluation, peer review, and presentation feedback) during this two-year period, form the foundation for this article to identify the essential components in developing GSW-specific interventions. 

Based on the global context of climate change, disasters, and other diverse crises, Karls and O’Keefe’s Person-in-Environment (PIE) theory has been adopted to guide the two authors’ critical reflection approach to examining the different levels of social work practice in disaster and non-disaster settings [6]. The critical reflection framework consists of three-level components, including micro-level: social work interventions that directly engage with service users (e.g., individuals, families, or small groups); mezzo-level: social work strategies that serve particular groups of clients at the agency and community levels (e.g., community-based social service agencies and community social development interventions); and macro-level: social work system-level approaches for advocacy, policymaking, and program development (e.g., social policy development and social work program design) [7,8]. These three-level social work interventions encourage both authors to identify the essential capacities that green social workers need to develop through in-class and field education. Consequently, this article is guided by the research question: what are the critical GSW-driven components associated with micro-, mezzo-, and macro-level social work interventions pertaining to supporting and promoting the individual and collective health and well-being of individuals, families, and communities affected by extreme events?

## 2. Green Social Work Research, Practice, and Education

Tragedies, occurring worldwide, caused by climate change, disasters, and other diverse crises and impacting almost all the societal dimensions (e.g., environmental, social, economic, political, and health), urgently call for social work practice to advocate for environmental justice and social justice for all inhabitants and co-inhabitants. In response, at home and abroad, social workers have been actively responding to these extreme events, reducing systemic vulnerabilities, promoting individual and collective health and well-being, and building resilience at the individual, family, community, and societal levels [3]. These efforts, understood as GSW interventions, further support the United Nations’ overarching plans for climate change adaptation and disaster risk reduction [9] and aim to achieve the UN’s 2030 Agenda for Sustainable Development [10]. Through portraying the landscape of GSW endeavors, this section will primarily focus on identifying the areas for improvement within GSW-specific education and training, in which this article is rooted.

### 2.1. Multi-Dimensional Influences and Compounded Impacts

Extreme events devastate almost all the societal sectors, for instance, destroying housing and critical infrastructural systems [11], suspending healthcare and social services [12], and disrupting economic growth [13]. These negative impacts not only cause compounded impacts on global citizens’ health and well-being, but also reversely reinforce the frequency, scope, and magnitude of extreme events and further increase inhabitants’ exposure to related hazards, weakening resilience and threatening sustainability at the individual, family, community, and societal levels [14,15,16]. GSW interventions require social workers to respond to these disaster-specific, multi-dimensional influences and compounded impacts [17]. More importantly, green social workers must identify and address the root causes of these influences and impacts, namely structural inequalities, such as poverty, health disparities, and food and natural resources security, and fundamentally advocate for environmental justice and social justice [18]. Historically, social workers have stood on the frontline, fighting these systemic injustices in our societal systems, and have advocated for human health and well-being in non-disaster scenarios [19,20]. Extreme events add an extra layer and increase the contextualized complexity of social work practice. Applying the knowledge, skills, and interventions obtained from the non-disaster settings to their service in disaster surroundings would enable social workers to continue supporting vulnerable and marginalized groups. A paucity of literature, however, addresses this knowledge mobilization in social work education and professional training. Social work professionals have a long history of engagement in supporting individuals, families, and communities (particularly those of the vulnerable and marginalized) affected by extreme events worldwide through the entire disaster cycle [21] and in non-disaster settings [22,23].

Specifically, social workers provide consulting services immediately after a disaster [24], map community-based resources and coordinate community-based agencies that address disaster survivors’ health and social needs during the post-disaster reconstruction and recovery stages [25], and empower the grassroots leadership in developing community-based mitigation strategies and pre-disaster preparedness plans [26]. These contributions encourage the integration of the GSW stream into the existing social work educational system. With over ten years of development, GSW remains marginalized in mainstream social work education in Canada and internationally [3]. Furthermore, in order to equip social workers with the capacity to address the disaster-specific, multi-dimensional influences and compounded impacts, pedagogical approaches need to enhance the social worker’s comprehension of the interconnections among different societal dimensions on the local residents. Then, social workers can better mobilize their expertise to support vulnerable individuals and families (micro-level), reconstruct affected communities and agencies (mezzo-level), and inform the policy/decision-making (macro-level). Post-secondary social work education experience is expected to strengthen students’ learning and cognitive aspects of adaptive capacity to effectively respond to these changing environments [27]; however, current GSW pedagogical explorations in Canada have not, so far, effectively addressed this component.

### 2.2. Multidisciplinary Interventions

The multi-dimensional influences of extreme events produce compounded impacts on dwellers’ physical health, mental health, and well-being. These complex health and well-being issues need to be addressed by integrated and interdisciplinary knowledge and strategies [28], which are aligned with the interdisciplinary nature of the social work profession, especially in the health and social care sectors and community settings [29,30]. For example, according to Maramaldi and colleagues [31], social workers in the healthcare sector indicate that interdisciplinary training could swiftly place them within a multidisciplinary healthcare team and enhance the healthcare quality that these social workers could offer their clients. This could be accomplished by exploring various patient-specific factors (e.g., home environment, beliefs, and culture). Through a social-work-engineering collaborative course in global community development, students from both disciplines could extend beyond disciplinary boundaries to engage different knowledge, skills, and culture-sensitive approaches to jointly solve real-world problems [32]. The upward number of global extreme events and their complexity of influences urgently require an interdisciplinary approach. However, the interdisciplinary component has been slowly developing in the GSW education and professional training. 

The long history of social work interprofessional collaboration in developing micro-, mezzo-, and macro-level interventions indicates that the interdisciplinary component should be reflected in GSW practice as well. Indeed, during the post-Lushan earthquake reconstruction, social workers collaborated with architects and urban planners, promoting the local skilled workers’ engagement in the post-earthquake reconstruction of housing and communities [33]. These micro- and macro-level interdisciplinary collaborations accelerated the post-earthquake recovery and stimulated the grassroots efforts to support the post-earthquake trauma relief and protect community-based cultural heritage [34]. After the EF4 tornado in Alabama (2019), Holmes and colleagues reported that social workers collaborated with anthropologists to employ an interdisciplinary policy analysis and political-economic critique approach, stimulate the macro-level dialogue, and identify the deep-seated causes of human and economic loss caused by the tornado [35]. Although these GSW practices powerfully demonstrate the need for social workers to engage with multidisciplinary professionals in disaster settings, GSW training to raise the students’ interdisciplinary awareness remains under-researched.

### 2.3. Multi-Stakeholder Engagement

Historically, social workers, in general, play a critical role in actively responding to extreme events by highlighting that the systemic inequities are root causes, exposing increasing individuals and communities to potential risks and hazards [36]. These inequities result in extreme events being more devastating to vulnerable and marginalized groups (e.g., children and youth, older adults, Indigenous, refugees, people experiencing homelessness, and people living with disabilities). Supporting these under-represented groups has been the core component of social work practice and the foundation for community development principles [37]. At the same time, as argued above, extreme events increase the complexity of committing to this mandate. Responsively, GSW interventions will fulfill this mission by contributing to the building of equal, inclusive, and diverse communities. In fact, social workers have been leading a bottom-up approach to coordinate various community-based stakeholders and agencies, collectively identify the different types of vulnerabilities (e.g., access to healthcare and social services and unorganized community resources), and develop community-driven solutions [38]. These social workers’ micro-and mezzo-level interventions, as community builders and organizers, not only accelerate the affected individuals’ and communities’ reconstruction and recovery [39], but also promote community development. Although community development is one of the major streams in the social work profession, social work students and trainees might not fully understand the importance of community development in the GSW stream. 

Disaster-driven community development requires social workers’ macro-level advocacy for political and program support [40]. Disaster-specific policies and programs serve as top-down strategies to guarantee multi-stakeholder engagement in community-centered disaster risk management and post-disaster activities [41]. Macro-level social work training associated with policy and program development as well as advocacy for policy changes have generally focused on the social service sector during the non-disaster settings [42]. Integrating GSW into the traditional social policy-related social work education and training realm will build the students’ policy and program analysis and development capacity. This will ensure these macro-level interventions are implanted according to climate change adaptation and disaster risk reduction mandates and better serve the affected individuals, families, and communities [43]. The evaluation of GSW-specific pedagogical outcomes associated with the macro-level social work practice, at this time, remains sparse.

In brief, as a nascent stream in the social work profession, GSW addresses the interconnections among various factors associated with global extreme events. GSW also develops a holistic approach that examines the diverse impacts on individuals, families, communities, and societies. These areas for improvement in GSW education and training in particular, social work education, and professional training in general call for pedagogical evidence and outcomes to apply multidisciplinary and multi-stakeholder engagement in disaster-driven community development. Accordingly, in response to the master research question presented above, this article employs a critical reflection approach to illustrate GSW-specific teaching, training, and learning experiences from both instructor/supervisor and student/trainee perspectives.

## 3. Materials and Methods

### 3.1. Teaching–Learning Interactions

This study, taking place through two academic years (September 2020 to April 2022) in the School of Social Work at Dalhousie University, Halifax, NS, Canada, focuses on the teaching–learning interaction between the two authors, namely a junior social work faculty member with research expertise in GSW and resilience, and a Master of Social Work (MSW) program student with rich practice specialization in child welfare. During the first academic year (September 2020 to April 2021), both authors featured an instructor-student relationship in an MSW-level required course (online), entitled Social Work Practice Research Methods. During this academic year, the multi-disaster societal background (see the societal context section below) encouraged the instructor to integrate the GSW component into weekly student engagement throughout this course. From September 2021 to April 2022, the two authors’ relationship became a supervisor–trainee relationship, when the student began engagement in research-based social work field education. As various disasters swiftly unfolded at home and abroad, these supervisor–trainee interactions continually concentrated on GSW-specific research projects in Canada and internationally. During this virtual field education, the trainee participated in a complete research development cycle for several GSW-specific research projects, including the activities of developing a new research project proposal, data collection and data analysis, drafting academic publications, and conducting related knowledge mobilization activities (e.g., conference presentations, invited talks, and guest presentation in MSW courses). During these two academic years, the two authors communicated through emails and virtual weekly meetings. Both authors’ social work-related expertise and partial demographic information, which inform their critical reflections, are presented in Table 1.

### 3.2. Multi-Disaster Societal Context

During the two-year teaching-learning period, the multi-disaster context regionally, nationally, and internationally formed an ideal platform for engaging GSW in teaching and training. This multi-disaster context features all three types of hazards, namely, natural hazards, technological hazards, and intentional and willful hazards. Notably, the global public health emergency of the COVID-19 pandemic, progressing from local zoonotic disease (a natural hazard) to an international catastrophe, profoundly impacted international communities, uncovering tremendous societal injustices, and jeopardized inhabitants and co-inhabitants’ physical health, mental wellness, and overall well-being [44,45,46]. Throughout Canada, climate change-induced natural hazards, such as annual wildfires, floods, extreme high and low temperatures, snowstorms, and hurricanes, further increased environmental and social injustices for vulnerable and marginalized groups and negatively affected their health and well-being [47,48,49]. Furthermore, technological hazards, such as lead water pipe and power outage [50,51], and willful hazards, including the 2020 Nova Scotia attacks [52] and Russia’s invasion of the Ukraine and refugees [53], directly and indirectly, have caused negative tolls on the health and well-being of residents and co-residents in Canada and beyond. These domestic and international extreme events propel international social workers to contextualize their micro-, mezzo-, and macro-level interventions within the community, regional, national, and international milieus, reflecting environmental justice, advancing social justice, and contributing to global citizen’s health and well-being [54,55].

### 3.3. Critical Reflection Oriented Data Curation

Critical reflection is an essential pedagogical tool, promoting students’ understanding and their in-situ practice in social work education and training in particular [5,56] and in health-related disciplines (e.g., nursing, medicine, and public health) in general [57,58,59]. Critical reflection also features a research method, connecting theory to practice with discursive depth [60]. During the two-year period, the instructor’s expertise and the multi-disaster context encouraged his pedagogical implementation of GSW interventions into social work teaching and training. The internal and external feedback regarding teaching and training efforts (see the data analysis section below) stimulated his critical reflections regarding pedagogical, research, and practice improvements. Furthermore, this two-year learning and training was the first time for the student to learn GSW knowledge and skills. In addition to the learning and field training engagement with the journal faculty member, her personal social work practice experience in the child welfare sector through this multi-disaster context urged her to apply the GSW-specific expertise she learned to re-examine her previous practice interventions, and identify the best strategies to support child welfare. The instructor’s and the student’s critical reflections formulate the primary data of this article.

The instructor and the student maintained weekly communication (e.g., emails and virtual meetings) throughout these two academic years. This weekly communication approach allowed them to share and critique each other’s reflections. This critique process inspired them to re-investigate their teaching–training–learning and research–practice interventions and explore the necessary GSW-specific knowledge and skills to improve their micro-, mezzo-, and macro-level practice. Moreover, the third-party feedback from other students in the MSW research methods course, from the field education facilitator’s evaluation (at the beginning, in the middle, and at the end of the placement), and from the audience of conference presentations, invited talks, and guest presentations in the research methods course, were all integrated into the weekly critique, further stimulating critical reflection and evaluation, and eventually promoting the richness of this critical-refection-based data curation. 

### 3.4. Health and Well-Being-Driven Data Analysis 

Both authors took notes on all the critical reflections during these two years. A content analysis approach was employed to thematically analyze these critical reflection notes. Since climate change and disaster threaten inhabitants and co-inhabitants’ health and overall well-being, health and well-being became the overarching theme that guided the entire data analysis. The overarching theme was supported by three-level social work practices (micro, mezzo, and macro). The two authors identified the codes regarding social work interventions that would enhance the residents’ health and well-being at the individual and family (micro), community and organization (mezzo), and policy and program (macro) levels. 

**Figure 1:**
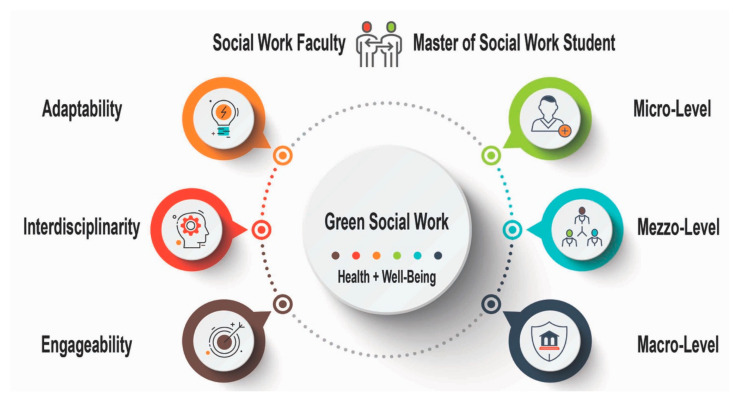
Data analysis structure. The three circles on the left (Adaptability, Interdisciplinarity, and Engageability) demonstrate the three themes that emerged in the data analysis. Under each theme, tri-level sub-categories (micro-level, mezzo-level, and macro-level, the three circles on the right) were developed to provide detailed supportive information from both authors’ critical reflections. These themes and subthemes are organized in a circle around the vital concept of GSW in this article, indicating the interconnections among these themes and subthemes.

The two authors collaboratively grouped the codes into three categories, reflecting the three essential components, namely adaptability, interdisciplinarity, and engageability, enhancing GSW practice in the global context of climate change, disasters, and other diverse crises. Under each category, each author presents their critical reflections on the three-level social work interventions (sub-themes) that are aligned with the GSW mandates of building healthy, resilient, and sustainable communities. These critical reflections not only directly address the health and well-being of residents but also illustrate related GSW interventions for the improvement of community-based service programs, service agencies’ roles in community development, and policy and program improvement, supporting the dwellers’ health and well-being. The data analysis structure is shown in Figure 1. 

## 4. Findings

GSW can be found in the full spectrum of social work interventions. This section will report the three essential components that emerged from the two authors’ (the instructor and the trainee) critical reflections associated with teaching–learning–practice. These critical reflections developed through micro-, mezzo-, and macro-level social work interventions feature crucial GSW components in supporting the health and well-being of inhabitants, families, and communities affected by climate change, disasters, and other diverse crises, as shown in Figure 1.

### 4.1. Adaptability

The swiftly unfolding global climate change, disaster, and diverse crises have been motivating social workers to be fully adaptable to quickly address the grassroots and community realities which are situated in related societal contexts (e.g., social, environmental, cultural, and political). This understanding assists social workers in advocating for policy and program improvement, supporting environmental and climate justice, and further advancing social justice. Social workers must make space for adequate reflection to absorb and understand this information.

#### 4.1.1. Instructor-Bridging Disaster and Non-Disaster Settings

Micro-level: The lack of GSW in social work curriculum and professional training in Canada limits students’ capacity to understand the integration of the influences of extreme events into addressing the health and overall well-being issues at the individual and family levels. Since the discipline of social work is rooted in social justice [61], connecting social justice and environmental/climate justice creates an accessible platform for students to be more mindful of environmental and climatological impacts on their client’s holistic health and well-being. This connection supports students in adapting their previous training regarding the effects of social injustice on human health and well-being and conceptualizing these impacts in the context of environmental and man-made crises. PIE-oriented reflection can help students examine their personal experiences to develop their comprehension. For instance, how would eco-anxiety worsen clients’ already weak health status? How would structural social injustice and environmental/climate pressures that a family may be experiencing collectively affect this family’s mental health and well-being? This training will build the students’ adaptability to utilize GSW interventions that promote grassroots health and well-being. 

Mezzo-level: The mezzo-level training focuses on increasing the students’ capacity in building community and/or agency’s capacity to prepare for, respond to, adapt to, and recover from disasters, contributing to collective health and well-being and building resilience [62]. Social workers’ community-based expertise enables them to swiftly connect clients with their community-specific resources in various settings (e.g., Indigenous, rural, and immigrant communities). The magnitude, scope, and intensity of extreme events determine that increasing numbers of communities that would be affected by the same crisis. Social workers’ community mapping expertise strengthens the partnership among stakeholders, agencies, and their resources within different communities, building collective interventions to protect the residents. Agency-based social work teams could also broaden cross-sector discussions, leveraging their agency-specific strengths to contribute to a more nuanced understanding of their clients’ individual and collective challenges and deepen their cross-sector collaboration in support of the affected communities. These two mezzo-level examples demonstrate the need to enlarge social workers’ adaptability from non-disaster to disaster scenarios. 

Macro-level: The macro-level training needs to emphasize students’ acknowledgment of the lived realities of individuals, families, and communities impacted by systemic injustices and extreme events. These two domains—systemic injustices and extreme events—jointly jeopardize individual and collective health and well-being. Responsively, social workers are vital players in advocacy for environmental/climate justice, resilience, and sustainability. This advocacy role motivates social workers to inform and improve related policies and programs to protect human rights in disaster settings, especially on behalf of vulnerable and marginalized groups. These ongoing efforts in advocacy and policy/program development and improvement support the social workers’ efforts toward protecting affected individuals, families, and communities.

#### 4.1.2. Student-Integrating Extreme Events’ Multi-Dimensional Influences on Residents’ Health and Well-Being

Micro-level: This trainee has supported the school children and their families’ health and well-being in elementary school settings. Her occupational environment, to some extent, limits her opportunities to fully appreciate the diverse social work roles in bolstering holistic client health associated with the broader scope of environmental and man-made crises. The GSW-specific in-class education and field training extended her understanding of social work interventions in the disaster landscape. The new knowledge has been encouraging the trainee to re-examine her interventions with the clients (school children and their families) affected by disasters, particularly during the 2015 European Refugee Crisis (at which time, refugee families were accepted into Canada and settled across the country), the 2016 Fort McMurray wildfires in Alberta, and the current COVID-19 pandemic. All these disasters, directly and indirectly, affected her clients’ physical health, mental health, and overall well-being. Social workers must swiftly adapt to this disaster-driven practice milieu by considering these disasters and related societal factors beyond their original occupational environment (in this case of elementary school settings). This adaptability also initiates a wide lens through which social workers can investigate interconnections among various health-related factors in developing disaster-informed interventions that advance the holistic consideration of inhabitants’ health and well-being.

Mezzo-level: General social work practice needs to be quickly adapted into unique community settings. As this trainee is a non-Indigenous social worker practicing in Indigenous communities, this adaptability is a critical part of her daily routine. The GSW training assists her in learning from Indigenous sustainability practices, building her adaptability to develop custom-specific strategies that promote the Indigenous community’s environmental health and well-being where she works. Indeed, through case studies of Indigenous activities that serve environmental protection and climate change mitigation, the student has discovered that many Indigenous communities have standard practices of respect and caring for mother earth, for example, cultural burning (Indigenous fire practices) to better the ecosystem [63]. This practice was once scrutinized by Western standards but has been recognized as an effective intervention to revitalize an ecosystem through safe practicing [64]. A healthy ecosystem, in turn, supports and fulfills inhabitants and co-inhabitants’ diverse needs, further contributing to their health and well-being because human health, animal welfare, and our shared environment are closely connected [65]. As this student is learning from the Indigenous community context models, obtaining the community-based knowledge, traditions, and skills enhances the social workers’ adaptability in general. It is an excellent means to advance the ecological, social, cultural, and health dimensions in community development. 

Macro-level: The GSW-specific training has been helping the trainee build a holistic approach to understanding the diverse factors (e.g., disasters and systemic injustices) that influence residents’ health and overall well-being, especially for vulnerable and marginalized groups. Unlike micro- and mezzo-level practices, where social work practitioners need to target the clients and communities’ unique requirements, applying an inclusive approach at the macro level informs the related policies and programs by protecting vulnerable and marginalized populations. The learning experience during the global public health emergency strengthens the student’s comprehension of the intersectionality of social injustices and the societal impacts on clients’ holistic health and well-being. For instance, Black, Indigenous, People of Colour (BIPOC) individuals statistically have shown higher rates of physical illness and death from COVID in North America compared to white counterparts [66]. COVID-19-driven racism increased for Asian Americans and Asian Canadians [67]. Social work anti-oppressive interventions have historically addressed social injustices and have supported vulnerable and marginalized groups [68]. Social workers’ expertise should be adapted to the contexts of environmental and man-made crises, continually perceiving how these crises specifically cause negative impacts on the health and well-being of the vulnerable and marginalized groups and addressing these issues through macro-level interventions.

### 4.2. Interdisciplinarity

Integrating the GSW component into social work education and training highlights the interdisciplinary social work interventions and promotes the effectiveness of the social work profession. For social workers to embrace interdisciplinarity, they must be reminded of the core emphasis of social work practice: social justice and equity. With this in mind, they can seek out like-minded professionals to partner with to bolster advocacy for clients, agency effectiveness, and equitable policies, as shown in the following reflections. 

#### 4.2.1. Instructor-Strengthening Students’ Interdisciplinary Disaster Efforts

Micro-level: Although the nature of social work discipline is interdisciplinary, practical modules to guide the interdisciplinary work at the primary micro-level practice level are still developing [69]. This professional deficit limits the social workers in applying interdisciplinary approaches to understand diverse factors associated with clients’ health and overall well-being. The full spectrum of disaster-specific influences on residents’ health and well-being propels social workers to work in multidisciplinary teams and develop interdisciplinary interventions. Accordingly, enhancing the students’ understanding of this range of impacts initiates the first step in building a collaborative foundation. Contextualizing influences on individuals and families within disaster surroundings improves the feasibility for students to embrace the root causes of these influences associated with different societal dimensions, including economic, social, cultural, and political. The interconnections among these dimensions trigger compounded impacts on individuals’ and families’ health and well-being, encouraging social workers to collaborate with other professionals to develop holistic interventions. 

Mezzo-level: GSW training opens a new opportunity for prospective social workers to go beyond their original practice scope and collaborate with other professionals to address environmental and climate injustices. Regarding mezzo-level practice, social work agencies can provide work-related opportunities for staff to engage with other community-based agencies outside the traditional social work scope. This interdisciplinary cooperation will create mutual benefits from each agency’s expertise and resources to identify the urgent and/or rooted issues and provide target solutions. GSW framework fosters a more nuanced understanding for the trainees towards the broader context of social work practice. This extended understanding encourages the students/trainees to re-examine their previous practice expertise and further apply a more holistic approach to addressing community inhabitants’ health and well-being. For instance, the trainee realized that in relation to climate-induced disasters, as one of the most vulnerable groups, children’s health and well-being are always negatively affected. She developed a disaster-aware approach to more actively put the GSW framework into her scope of practice in counseling those inhibited by eco-anxiety. She also trained other child welfare workers in her agency and other agencies to equip the staff to become “disaster responders,” prepared to respond to environment-related crises, and be aware of environmental injustice. The multifaceted influences on child welfare motivate her to frequently communicate with other professionals (e.g., psychologists and environmental sociologists) to develop interdisciplinary strategies.

Macro-level: Most social workers, who practice in the climate change and disaster field, focus mainly on the micro- and mezzo-level interventions. Since promoting environmental and climate justice necessitates macro-level program and policy support, the in-class social policy training is specific to social service policies and programs, where the climate change and disaster components may not continually be effectively developed. Hence, GSW-specific macro-level practice encourages the students to collaborate with professionals from other sectors to advocate for policy and program improvement [3]. Furthermore, the suggested educational and training opportunities should cultivate more macro-level-oriented social workers, who will integrate different societal dimensions to develop interdisciplinary approaches to address the climate change and disaster issues through policy and educational program improvement [70].

#### 4.2.2. Student-Raising the Awareness of GSW-Specific Interdisciplinary Engagement

Micro-level: Micro-level social work practice frequently collaborates with various professionals and stakeholders from diverse backgrounds. In fact, while working in an elementary school, the student partnered with teachers, administrative staff, and other support workers (e.g., psychologists and speech pathologists). The immense value of interdisciplinary work is that each profession can play to its strengths. Unlike the psychologist’s one-to-one consulting, school social workers support the students throughout their in-school and out-of-school time. This engagement with students strengthens the social workers’ comprehension of the triangulation of emotional, psychological, and social well-being and their overlapping influence on student mental health and overall well-being [71]. GSW training broadens the student’s acknowledgment of the triangulation and interconnected influences in the context of extreme events. Accordingly, the student brought the unique social work perspective emphasizing crisis-specific social and emotional well-being into the partnership with the psychologist to improve their mental health service. This interdisciplinary collaboration effectively addressed students’ unique needs in consideration of their holistic health. 

Mezzo-level: GSW-specific training increases students’ awareness of the multi-layers of interdisciplinary mezzo-level cooperation. Social work agencies focus on supporting community members’ full spectrum of needs, including holistic wellness, while liaising with public, private, and not-for-profit sectors. In non-disaster settings, social workers can collaborate with certain agencies, targeting the specific needs of local residents. However, as with the current pandemic, all the societal dimensions are devastated; this calls for strategical interdisciplinary mezzo-level partnerships among diverse stakeholders. In a project partnering with Indigenous communities to learn more about their COVID-driven public health responses, the student highlighted the need for social workers to cooperate with governing bodies, Indigenous elders, health and social care service agencies, and cultural and recreation sectors to develop culturally sensitive approaches that would identify the strengthens and weaknesses of traditionally Indigenous public health efforts. This experience also formulated the student’s perception regarding the transferability of the social work-specific knowledge and skills, which could be utilized and even advanced by other groups and organizations.

Macro-level: Before this learning, the student felt that macro-level social work interventions toward climate change, disaster, and diverse crises were always “far away” and inaccessible due to her school-based occupational practice settings. After this training, the student realized the social worker’s critical role in climate change adaptation and disaster risk reduction by advocating environmental justice and social justice and enhancing the residents’ health and well-being [72]. The potency of social work research, interacting with agents from different realms, especially government and academia, informs policy/decision-making and program improvement. Furthermore, macro-level social work practices are required to apply transferable social work knowledge and skills while engaging with policymakers and stakeholders from various sectors. These multidisciplinary conversations enact change and policies to better holistic health and well-being for vulnerable individuals and marginalized groups.

### 4.3. Engageability

As indicated above, the global context of climate change, disasters, and other diverse crises calls for a preventative practice, actively engaging the community residents, public, private, and not-for-profit agencies, and other stakeholders in disaster-driven community development, supporting the residents’ health and well-being. The instructor and the student’s critical reflections highlight micro-, mezzo-, and macro-level GSW understanding and interventions, promoting community engagement in both non-disaster and disaster settings. 

#### 4.3.1. Instructor-Preparing Students’ GSW-Specific Community-Based Expertise

Micro-level: GSW-specific community-based expertise allows students to swiftly connect residents with their required services and resources. This community asset mapping strategy promotes diverse community-based stakeholders’ engagement in community development [73]. Social workers also serve on the boards of community-based agencies. This function deepens the students’ understanding and allows trainees to utilize their knowledge and expertise to enhance the agencies’ services, better addressing the residents’ unique requirements. Since disaster affects almost all the societal dimensions, therefore, community asset mapping is transferable to disaster settings, enabling social workers to engage various stakeholders in disaster efforts and better respond to affected individuals and families. 

Mezzo-level: The community asset mapping expertise can be applied at the mezzo level so that social workers can partner with community-based agencies to promote community engagement [74]. GSW-specific education and training enable students to leverage the “service delivery” and “knowledge expertise” inventories of agencies to better identify areas that need improvement at the community level and to enhance the community’s pre-disaster preparedness. For instance, community-based pre-disaster preparedness has been slowly developing in Canada, providing social workers various opportunities to support disaster mitigation efforts. Social workers can facilitate agencies’ community outreach activities to educate the general public and raise their disaster preparedness awareness. Social workers can help these agencies improve their disaster and emergency response planning (e.g., building organizational emergency response plans and providing agency-wide staff-specific disaster preparedness training). These efforts encourage cross-sector collaboration among agencies and professionals to be ready to serve local inhabitants when disasters hit.

Macro-level: The social worker’s advocacy role in integrating environmental justice and climate justice into community development further informs policy and program improvement and development [75,76]. GSW learning and training require that students be mindful of the marks of healthy community engagement, supported by continuous and consistent policy and program support, during both disaster and non-disaster scenarios, and consistently promote that the lessons learned from the disaster scenarios be reflected in the policy and program adjustment. Social work educators must assert that community engagement and development are among the fundamental components of social work profession, that support almost all types of social work practice and individual and collective health and well-being. GSW-specific training can be provided for prospective community-based social workers to enhance their roles in community engagement regarding supporting residents’ health and well-being in disaster and non-disaster settings.

#### 4.3.2. Student-Promoting Multi-Stakeholder Engagement in Disaster-Driven Community Development

Micro-level: The trainee used to believe that community development was a ‘niche’ social work practice. GSW-specific practice always highlights community residents’ collaborative engagement in post-disaster community reconstruction and recovery [77]. This stimulates the trainee to re-evaluate how social workers’ community-based expertise could empower grassroots efforts to promote community development in disaster settings. Specifically, the trainee recalled her previous experience in coordinating summer activities and camps for school children. This job established her connections with different community members and agencies, such as local bakeries and recreation facilities (e.g., swimming pools and indoor playgrounds). This network enhanced the school children’s summer experiences and supported their mental and health well-being. The trainee discovered that these community stakeholders she contacted actually desired to ‘give back’ to their communities, especially by helping vulnerable dwellers but did not know how to actualize their well-wishes. This highlights the value of community-based social work interventions and the need for social work practitioners and other social service workers to access and motivate these community-based assets. When transferring into disaster settings, the established networks and connections could swiftly engage diverse stakeholders in disaster-driven community development.

Mezzo-level: Working in the elementary school setting, the trainee coordinates non-profit agencies to contribute to after-school student programming, including emergency eviction instructions provided by the local police stations, refugee settlement service agencies’ free breakfast programs and translation programs for newcomer families, and the provincial mental health agencies’ consulting service. These programs not only address the school students and their families’ immediate needs (e.g., emergency response skills, food, and parents’ re-employment efforts) but also contribute to students’ physical health (e.g., nutrition and physical activity) and mental health needs (e.g., opportunity to extend their social networks and the general public’s education of holistic wellness). The cross-sector community engagement during the non-disaster period builds the community’s coping capacity for future extreme events. Social work endeavors can enhance the schools’ community hub position for community development, supporting the students, their families, and other extended community members’ holistic wellness. 

Macro-level: Governmental structures and related interventions (e.g., funding and resource allotment) primarily impact community engagement. For example, reducing school social worker positions and downsizing or eliminating non-profit agencies’ programming can threaten a school’s community hub role discussed above, further jeopardizing community-based social services. This has a particularly negative impact on vulnerable community residents. Community-based social work efforts advocate community engagement and empowerment in preparing communities to respond to potential climate-related and other disasters. Hence, this advocacy role of social workers in non-disaster settings can inform the related governmental interventions to be more aware of the significance of community engagement and can strengthen the community’s coping capacity for potential disasters, while contributing to healthy, resilient, and sustainable community development.

## 5. Discussion

As climate change, disasters, and other diverse crises continue to ravage natural resources, human settlements, inhabitants’ livelihoods, and inhabitants and co-inhabitants’ health and well-being, the three critical components developed from two social workers’ critical reflections should be considered in order to enhance the three-level GSW interventions and promote individual and collective health and well-being. Based on the three crucial components developed above, in this section, the two authors will synthesize the GSW-specific essential capacities in order to advance GSW education, training, and practice through the three-level social work interventions. It is worth mentioning that these GSW-specific capacities shed light on developing related pedagogical approaches to engage climate change, disasters, and other crises-driven components in other health care and social care professional fields, promoting multidisciplinary multi-stakeholder engagement to advance resilience and sustainability.

In the non-disaster circumstance, micro-level GSW interventions contribute to residents’ health and well-being by connecting the residents with related resources and services. This capacity forms the foundation for social workers to adapt their expertise from non-disaster scenarios to disaster settings, where they actively serve individuals and families impacted by catastrophic events. When extreme events disrupt various societal dimensions and trigger compounded influences on disaster survivors’ health and well-being, social workers must be swiftly adapted into this multi-system influence context and collaborate with professionals from multidisciplinary backgrounds to develop resident-driven interdisciplinary solutions [78]. This interdisciplinary cooperation connects different community-based networks and resources, enhances multi-stakeholder community engagement, and eventually contributes to healthy, resilient, and sustainable community development [77]. The benefits from the positive community development, in turn, build the individual and family’s coping capacity for potential extreme events. 

Community is the foundation that unites all inhabitants’ collaborative response to extreme events, promoting collective health and well-being; community-based agencies provide related resources, services, and networks to enable the foundation (community)’s mandates [79]. Bridging communities and agencies, social workers could support both sectors in building their coping capacity during pre-disaster preparedness and disaster mitigation stages to protect their residents/clients when disaster hits, physically, socially, emotionally, psychologically, and economically [80]. All these different health-related dimensions require interdisciplinary cooperation. Hence, mezzo-level GSW practice should promote multidisciplinary multi-stakeholder engagement in disaster-driven community and agency development to reduce extreme events’ negative influences on individual and collective health and well-being. 

In non-disaster settings, macro-level social work strategies advocate for inclusive policies and programs to protect everyone when disasters hit [81]. Since vulnerable and marginalized populations are always more detrimentally affected by extreme events, social workers should provide a particular focus on these groups in disaster settings. Furthermore, supporting the interdisciplinary collaboration enhances social workers’ capacity to inform the related policies and programs that could improve multi-stakeholder community development. GSW requires social workers to advocate environmental and social justice in policy and program development [75]. These policies and programs, in turn, should guarantee the education of more social workers and other professionals who can apply a holistic approach to address the individual and collective health and well-being. 

## 6. Conclusions

This article identifies three critical components of GSW through two authors’ critical reflections. Integrating the knowledge and skills base of GSW within the core social work professional education and training is vital for cultivating prospective green social workers [82]. GSW training assists students in adapting the traditional social work knowledge and interventions from non-disaster scenarios into disaster settings and applies a holist approach to consider multi-factor influences on residents’ health and well-being. The multi-dimensional consequences of extreme events raise the awareness of the importance of the interdisciplinary nature of social work. GSW education builds students’ interdisciplinary capacity so that these potential social workers can work in multidisciplinary professional teams and utilize interdisciplinary interventions to support affected individuals, families, and communities when disaster hits. This multidisciplinary cooperation empowers the social workers’ community-based expertise to promote multi-stakeholder engagement in disaster-driven community development and enhance the individual and collective health and well-being at the individual, family, community, and societal levels. 

The upward tendencies of global climate change, disasters, and other diverse crises have been urgently calling for GSW interventions. This article qualitatively highlights the three GSW practice components, determined from the two authors’ critical reflections and evaluations. Understandably, the authors’ critical reflections and evaluations are primarily influenced by their personal experiences and backgrounds (including the demographic variables), which have not been effectively examined in this article. Future studies could draw from a larger sampling size, apply different research methods (e.g., quantitative and mixed-method), and investigate and evaluate the essential components and evidence-based strategies that utilize GSW-specific pedagogical innovations. These efforts will promote GSW-specific education and professional training, and more importantly, integrate environmental and climate justice into social work research, education, and practice, advancing social work advocacy for social justice and eventually building healthy, resilient, and sustainable communities for all the inhabitants and co-inhabitants. Furthermore, global extreme events call for an all-inclusive approach to engage all stakeholders [83]. GSW also suggests developing related pedagogical innovations to engage extreme event-driven components in different disciplines. These approaches will eventually establish multidisciplinary professional teams to collaborate with various stakeholders in public, private, and not-for-profit sectors, building resilient and sustainable societies for all inhabitants and co-inhabitants. 

## Figures and Tables

**Table 1:** Social work expertise and demographic information.

Social Work Profession		Junior Faculty	MSW Student
Role in Social Work		Researcher	Practitioner
Role in Social Work Course		Instructor	Student
Role in Social Work Practicum		Supervisor	Trainee
Social Work Research/Practice Field		Green Social Work, Climate Change Adaptation and Disaster Risk Reduction, and Resilience	Children Welfare
Social Work Practice Location		International and Canada	Alberta, Canada
Demographic variables	Gender and Sexual Minority	Yes	Yes
	Immigration Status	Yes	No
	Visible Minority	Yes	No

## Data Availability

Not applicable.

## References

[1] Canadian Association of Social Work (CASW) What Is Social Work. https://www.casw-acts.ca/en/what-social-work.

[2] Dominelli L. (2012). Green Social Work: From Environmental Crises to Environmental Justice.

[3] Wu H. (2021). Integration of the Disaster Component into Social Work Curriculum: Teaching Undergraduate Social Work Research Methods Course during COVID-19. Br. J. Soc. Work..

[4] Dorlet J. (2019). Rebuilding Lives Post-Disaster.

[5] Ferguson H. (2018). How social workers reflect in action and when and why they don’t: The possibilities and limits to reflective practice in social work. Soc. Work. Educ..

[6] Karls J.M., O’Keefe M.E. (2008). Person-in-Environment System Manual.

[7] Social work guide. Micro vs. Mezzo vs. Macro Social Work. https://www.socialworkguide.org/resources/micro-vs-mezzo-vs-macro-social-work/.

[8] Conrad-Amlicke G. Understanding Micro, Mezzo and Macro Social Work Practice. https://www.socialworkers.org/Careers/NASW-Career-Center/Explore-Social-Work/Understanding-Micro-Mezzo-and-Macro-Social-Work-Practice.

[9] United Nations Resolution Adopted by the General Assembly on 25 September 2015. https://www.un.org/ga/search/view_doc.asp?symbol=A/RES/70/1&Lang=E.

[10] The 2030 Agenda for Sustainable Development. https://sdgs.un.org/sites/default/files/publications/21252030%20Agenda%20for%20Sustainable%20Development%20web.pdf.

[11] Wu H. (2021). When Housing and Communities Were Delivered: A Case Study of Post-Wenchuan Earthquake Rural Reconstruction and Recovery. Sustainability.

[12] Wu H., Karabanow J. (2020). COVID-19 and beyond: Social work interventions for supporting homeless populations. Int. Soc. Work..

[13] Carleton T.A., Hsiang S.M. (2016). Social and economic impacts of climate. Science.

[14] Wu H. (2020). Airdropped urban condominiums and stay-behind elders’ overall well-being: 10-year lessons learned from the post-Wenchuan earthquake rural recovery. J. Rural. Stud..

[15] Wu H., Etienne F., Letcher T.M. (2021). Effect of climate change on food production (animal products). A Comprehensive Study of Physical, Social, and Political issues.

[16] Peek L., Tobin J., Adams R.M., Wu H.R., Mathews M.C. (2020). A Framework for Convergence Research in the Hazards and Disaster Field: The Natural Hazards Engineering Research Infrastructure CONVERGE Facility. Front. Built Environ..

[17] Dominelli L. (2013). Environmental justice at the heart of social work practice: Greening the profession. Int. J. Soc. Welf..

[18] Drolet J., Wu H.R., Taylor M., Dennehy A. (2015). Social Work and Sustainable Social Development: Teaching and Learning Strategies for ‘Green Social Work’ Curriculum. Soc. Work. Educ..

[19] Dominelli L. (2021). A green social work perspective on social work during the time of COVID-19. Int. J. Soc. Welf..

[20] Banks S., Cai T., Jonge E., Shears J., Shum M., Sobocan A.M., Strom K., Truell R., Uriz M., Weinberg M. Ethical Challenges for Social Workers during COVID-19: A Global Perspective. https://www.ifsw.org/wp-content/uploads/2020/07/2020-06-30-Ethical-Challenges-Covid19-FINAL.pdf.

[21] Drolet J., Alston M., Dominelli L., Ersing R., Mathbor G., Wu H. (2015). Women rebuilding lives post-disaster: Innovative community practices for building resilience and promoting sustainable development. Gend. Dev..

[22] Richmond M.E. (1917). Social Diagnosis.

[23] Addams J. (1922). Peace and Bread in Time of War.

[24] Curry A., Simpson S. (2021). Cross-training Social Workers to Work in a Psychiatric Emergency Service during the COVID-19 Pandemic. Acad. Psychiatry.

[25] Drolet J., Ersing R., Dominelli L., Mathbor G., Alston M., Mathbor G., Huang Y., Wu H. (2018). Rebuilding lives and communities post-disaster: A case study on migrant workers and diversity in the U.S. Aust. Soc. Work..

[26] Das A. (2020). Social Work, Disasters and Communities-Challenging the Boundaries of the Profession. Br. J. Soc. Work..

[27] Walker S.E., Bruyere B.L., Zarestky J., Yasin A., Lenaiyasa E., Lolemu A., Pickering T. (2021). Education and adaptive capacity: The influence of formal education on climate change adaptation of pastoral women. Clim. Dev..

[28] Clover D.E., Sanford K., Inman P., Robinson D.L. (2014). Introduction. University Engagement and Environmental Sustainability.

[29] Lennon-Dearing R., Florence J., Garrett L., Click I.A., Abercrombie S. (2008). A rural community-based interdisciplinary curriculum: A social work perspective. Soc. Work. Health Care.

[30] Bronstein L., Mizrahi T., Korazim-Korosy Y., McPhee D. (2010). Interdisciplinary collaboration in social work education in the USA, Israel and Canada: Deans’ and directors’ perspectives. Int. Soc. Work..

[31] Maramaldi P., Sobran A., Scheck L., Cusato N., Lee I., White E., Cadet T.J. (2014). Interdisciplinary Medical Social Work: A Working Taxonomy. Soc. Work. Health Care.

[32] Gilbert D.J. (2014). Social Work and Engineering Collaboration: Forging Innovative Global Community Development Education. J. Soc. Work. Educ..

[33] Wu H. (2018). Promoting public interest design: Transformative change toward green social work during post-Lushan earthquake reconstruction and recovery in Sichuan, China. Handbook of Green Social Work, Dominelli, L., Ku, H.B., Nikku, B.R., Eds..

[34] Wu H.R., Hou C.P. (2019). Utilizing co-design approach to identify various stakeholders’ roles in the protection of intangible place-making heritage The case of Guchengping Village. Disaster Prev. Manag..

[35] Holmes T.J., Mathias J., McCreary T., Elsner J.B. (2021). What’s the problem with disaster? Anthropology, social work, and the qualitative slot. Qual. Soc. Work..

[36] Schibli K., Canadian Association of Social Workers Position Statement 2020. https://www.casw-acts.ca/files/documents/SW_and_Climate_Change_Final_PDF.pdf.

[37] Rowlands A. (2013). Disaster Recovery Management in Australia and the Contribution of Social Work. J. Soc. Work. Disabil. Rehabil..

[38] Wu Y.F., Wen J., Wei H. (2019). Community-centred’ as an integrated model for post-disaster social work-The case of earthquake-stricken Ludian, China. Asia Pac. J. Soc. Work. Dev..

[39] Ku H.B. (2015). Post-disaster Community Development in Rural Sichuan, China. J. Rural. Community Dev..

[40] Sim T., He M.Y., Dominelli L. (2022). Social Work Core Competencies in Disaster Management Practice: An Integrative Review. Res. Soc. Work. Pract..

[41] Pandey C.L. (2019). Making communities disaster resilient Challenges and prospects for community engagement in Nepal. Disaster Prev. Manag..

[42] Dominelli L. (2017). Climate Change Rethinking the Local for Policy and Practice.

[43] Dominelli L. (2019). Green Social Work, Political Ecology and Environmental Justice.

[44] Robinson K., Briskman L., Mayar R. (2021). Disrupting Human Rights: A Social Work Response to the Lockdown of Social Housing Residents. Br. J. Soc. Work..

[45] Lee E., Johnstone M. (2021). Resisting politics of authoritarian populism during COVID-19, reclaiming democracy and narrative justice: Centering critical thinking in social work. Int. Soc. Work..

[46] Wu H.R., Mackenzie J. (2021). Dual-Gendered Leadership: Gender-Inclusive Scientific-Political Public Health Communication Supporting Government COVID-19 Responses in Atlantic Canada. Healthcare.

[47] Keith E. Experts Warn of an Above-Average Hurricane Season & a “Higer Risk” for Atlantic Canada. https://www.narcity.com/hurricane-season-2021-will-be-above-average-especially-in-atlantic-canada.

[48] Kulkarni A. A Look Back at the 2021 BC Wildfire Season. https://www.cbc.ca/news/canada/british-columbia/bc-wildfires-2021-timeline-1.6197751.

[49] Chung E. 10 Weather Stories that Made 2021 a Year Like no Other. https://www.cbc.ca/news/science/top-10-weather-stories-2021-1.6288139.

[50] Cooke A. Thousands Left Without Power after Snow Plow Hits Power Pole in Sackville. https://globalnews.ca/news/8658619/lower-sackville-snow-plow-power-pole/.

[51] Gómez P. Hazardous Levels of Lead in Canadian Tap Water. https://fcpp.org/2020/01/12/hazardous-levels-of-lead-in-canadian-tap-water/.

[52] Collins S. What We Know about a Mass Shooting in Nova Scotia, Canada. https://www.vox.com/world/2020/4/19/21227349/mass-shooting-nova-scotia-canada-enfield-portapique.

[53] Government of Canada Canada to Welcome Those Fleeing the War in Ukraine. https://www.canada.ca/en/immigration-refugees-citizenship/news/2022/03/canada-to-welcome-those-fleeing-the-war-in-ukraine.html.

[54] Harrikari T., Romakkaniemi M., Tiitinen L., Ovaskainen S. (2021). Pandemic and Social Work: Exploring Finnish Social Workers’ Experiences through a SWOT Analysis. Br. J. Soc. Work..

[55] Henley L.J., Henley Z.A., Hay K., Chhay Y., Pheun S. (2021). Social Work in the Time of COVID-19: A Case Study from the Global South. Br. J. Soc. Work..

[56] Theobald J., Gardner F., Long N. (2017). Teaching Critical Reflection in Social Work Field Education. J. Soc. Work. Educ..

[57] Bagheri M., Taleghani F., Abazari P., Yousefy A. (2019). Triggers for reflection in undergraduate clinical nursing education: A qualitative descriptive study. Nurse Educ. Today.

[58] Dundas K.J., Hansen V., Outram S., James E.L. (2017). A Light Bulb Moment in Understanding Public Health for Undergraduate Students: Evaluation of the Experiential This Is Public Health Photo Essay Task. Front. Public Health.

[59] Winkel A.F., Yingling S., Jones A.A., Nicholson J. (2017). Reflection as a Learning Tool in Graduate Medical Education: A Systematic Review. J. Grad. Med. Educ..

[60] Fraser M., Wotring A., Green C., Eady M. (2022). Designing a framework to improve critical reflection writing in teacher education using action research. Educ. Action Res..

[61] Segal E.A., Wagaman M.A. (2017). Social Empathy as a Framework for Teaching Social Justice. J. Soc. Work. Educ..

[62] Dominelli L. (2015). The opportunities and challenges of social work interventions in disaster situations. Int. Soc. Work..

[63] Wickham S., Trant A., Davis E., Hoffman K. (2021). How Indigenous Cultural Burning Practices Benefit Biodiversity. https://thenarwhal.ca/wildfires-indigenous-cultural-burning-biodiversity/.

[64] Okoyomon A. (2021). Good Fire: Revitalizing Cultural Burning. https://www.scienceworld.ca/stories/good-fire-revitalizing-cultural-burning/.

[65] Mackenzie J.S., Jeggo M. (2019). The One Health Approach-Why Is It So Important?. Trop. Med. Infect. Dis..

[66] Wallis C. (2020). Why Racism, Not Race Is a Risk Factor for Dying of COVID-19. https://www.scientificamerican.com/article/why-racism-not-race-is-a-risk-factor-for-dying-of-covid-191/.

[67] Wherry A. (2020). One Country, Two Pandemics: What COVID-19 Reveals about Inequality in Canada. https://www.cbc.ca/news/politics/pandemic-covid-coronavirus-cerb-unemployment-1.5610404.

[68] Rogers J. (2012). Anti-Oppressive Social Work Research: Reflections on Power in the Creation of Knowledge. Soc. Work. Educ..

[69] Bronstein L., Kovacs P., Vega A. (2007). Goodness of fit: Social work education and practice in health care. Soc. Work. Health Care.

[70] Gum N. (2022). Environmental Policy and Social Work: A Call to Action. Health Soc. Work..

[71] Kinman G., Grant L. (2011). Exploring Stress Resilience in Trainee Social Workers: The Role of Emotional and Social Competencies. Br. J. Soc. Work..

[72] Boetto H., Bell K., Ivory N. (2021). Disaster Preparedness in Social Work: A Scoping Review of Evidence for Further Research, Theory and Practice. Br. J. Soc. Work..

[73] Lightfoot E., McCleary J.S., Lum T. (2014). Asset Mapping as a Research Tool for Community-Based Participatory Research in Social Work. Soc. Work. Res..

[74] Parker J., Crabtree S.A. (2016). Part of the soluion or part of the problem? Reflections on teaching participatory asset mapping. Community Dev. J..

[75] Dominelli L. (2011). Climate change: Social workers’ roles and contributions to policy debates and interventions. Int. J. Soc. Welf..

[76] Peeters J. (2012). A comment on Climate change: Social workers’ roles and contributions to policy debates and interventions’. Int. J. Soc. Welf..

[77] Sim T., Liu Y., Li S.J. (2017). Working together: Developing disaster risk reduction first aid training in a post-earthquake Chinese context. J. Soc. Work..

[78] Drolet J.L., Lewin B., Pinches A. (2021). Social Work Practitioners and Human Service Professionals in the 2016 Alberta (Canada) Wildfires: Roles and Contributions. Br. J. Soc. Work..

[79] Ashida S., Zhu X., Robinson E.L., Schroer A. (2018). Disaster preparedness networks in rural Midwest communities: Organizational roles, collaborations, and support for older residents. J. Gerontol. Soc. Work..

[80] Karabanow J., Bozcam E.S., Huges J., Wu H. (2021). Lessons learned: COVID-19 and individuals experiencing homelessness in global context. Int. J. Homelessness.

[81] Wu H. (2022). Mass email risk communication: Lessons learned from the COVID-19-triggered campus-wide eviction in Canada and the United States. PLoS ONE.

[82] Rowlands A. (2013). Social Work Training Curriculum in Disaster Management. J. Soc. Work. Disabil. Rehabil..

[83] United Nations Sendai Framework for Disaster Risk Reduction 2015–2030. https://www.undrr.org/publication/sendai-framework-disaster-risk-reduction-2015-2030.

